# Spontaneous soft tissue un-puckering of a surgical neck of humerus fracture: a case report

**DOI:** 10.1093/jscr/rjab430

**Published:** 2021-09-27

**Authors:** Alistair Olive, Jonathan Jessup, Karanjit Mangat

**Affiliations:** Warwick Medical School, Medical School Building, University of Warwick, Coventry, UK; Warwick Medical School, Medical School Building, University of Warwick, Coventry, UK; Orthopaedic Department, Warwick Hospital, South Warwickshire NHS Foundation Trust, Warwick, UK

## Abstract

Fractures of the proximal humerus are commonly seen, and skin puckering is a rare clinical sign associated with such injuries. It is a relative indication to proceed with urgent surgical management due to the pending threat to soft tissue viability. To the best of our knowledge, this is the first report to describe a spontaneous un-puckering of the soft tissues in such an injury and it resulted in subsequent successful non-operative treatment.

## INTRODUCTION

The Pucker sign is a rarely seen clinical manifestation of proximal humeral fractures [1]. The sign has been scarcely reported in adults but is more widely reported in the upper limb fractures of children [2–5].

Puckering of the skin in fractures of the proximal humerus occurs as a result of fracture fragments ‘buttonholing’ through the deltoid muscle and into the dermis. This causes soft tissue tethering and interposition into the site of fracture. The aforementioned process can compromise local neurovasculature and increase the risk of soft tissue necrosis. Surgical management is almost always indicated for such injuries in order to prevent the loss of soft tissue integrity.

To the best of our knowledge, this is the first description of spontaneous ‘un-puckering’ of such injuries and resulted in the patient undergoing successful non-operative treatment.

## CASE REPORT

A 78-year-old Caucasian male was admitted following a collapse and loss of consciousness. His past medical history included a permanent pacemaker due to bifascicular block. Appropriate investigations were undertaken to identify the cause of his symptoms.

**Figure 1:**
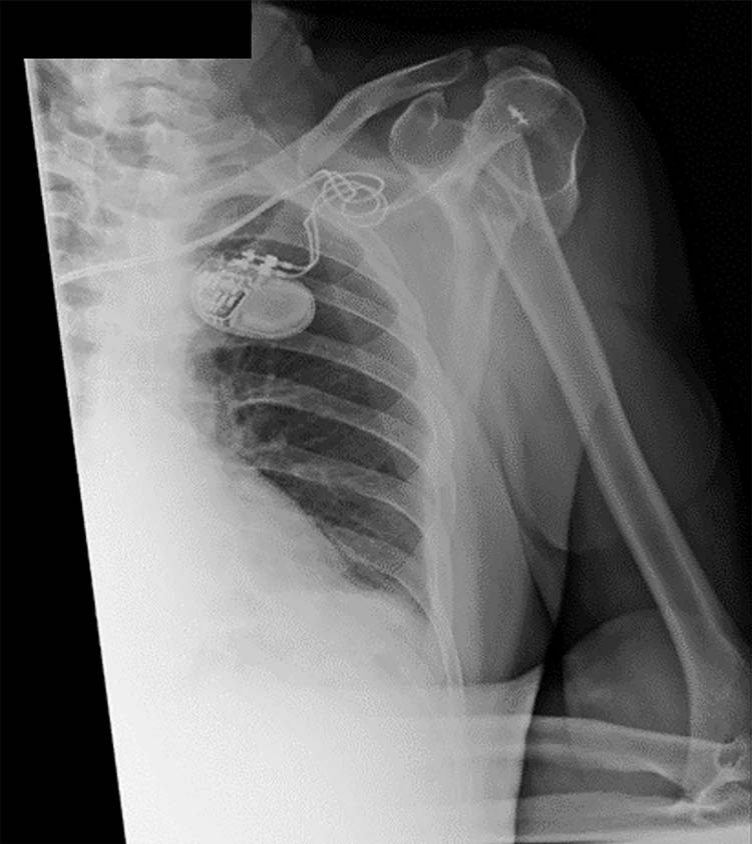
Antero-posterior view X-ray taken on day of admission, showing a left surgical neck of humerus fracture with anterior displacement of the proximal humeral shaft.

**Figure 2:**
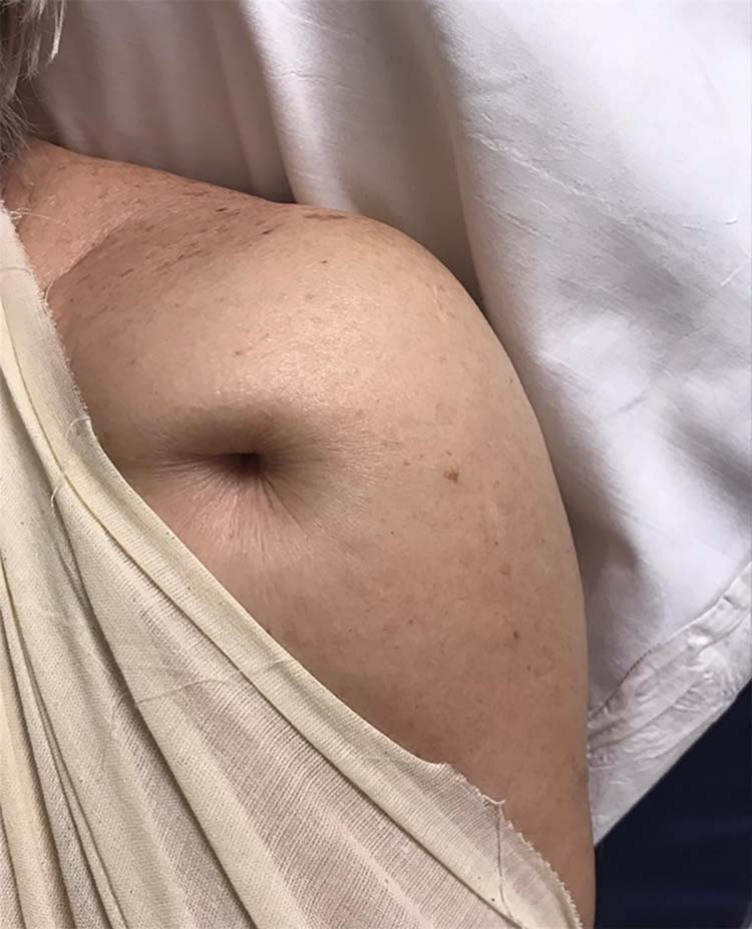
Clinical picture showing ‘Pucker sign’ on the left shoulder (anterior view).

Examination and imaging of the patient revealed that during the fall, he had sustained a left surgical neck of humerus fracture (Fig. 1). Significant skin puckering was noted on the anterior aspect of the left shoulder over the fracture site, otherwise the arm was neurovascularly intact (Fig. 2). He was immediately reviewed by the orthopaedic team who offered surgical fixation of the fracture on the following morning, which was dependent on an appropriate anaesthetic assessment. The patient was in agreement and consented to proceed.

**Figure 3:**
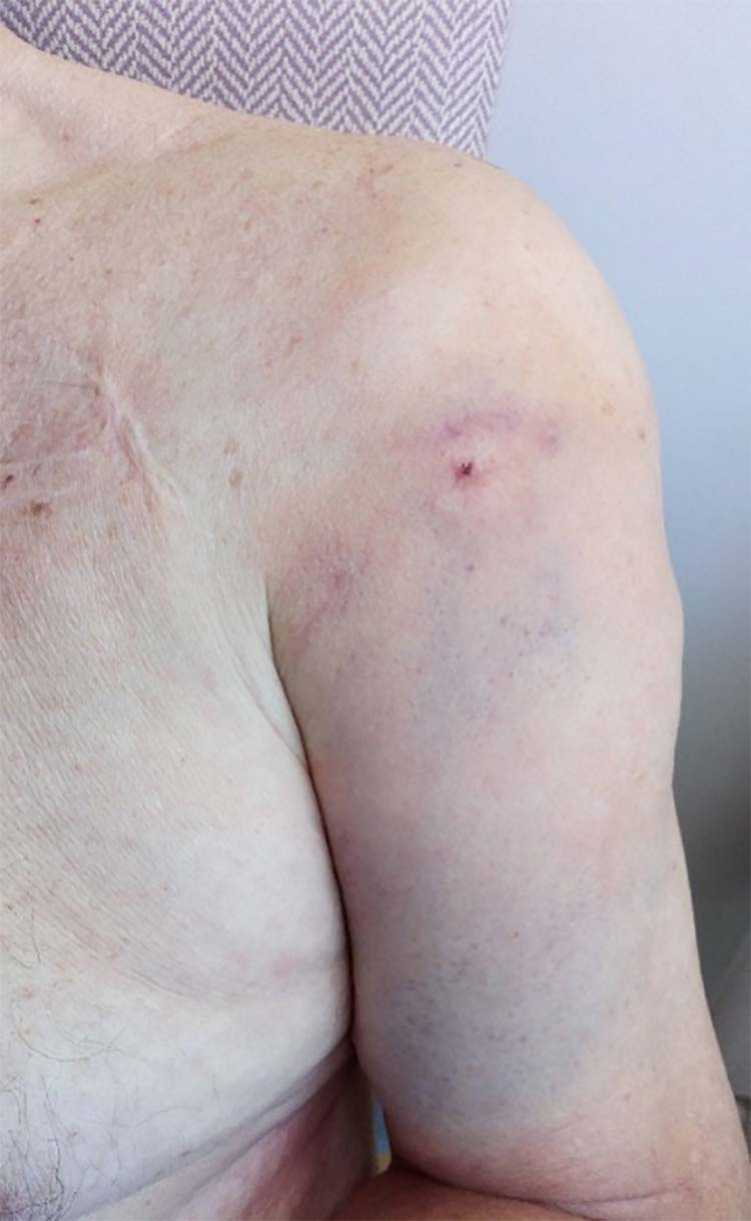
Clinical picture showing un-puckered left shoulder and localized ecchymoses of the anterior arm (anterior view).

**Figure 4:**
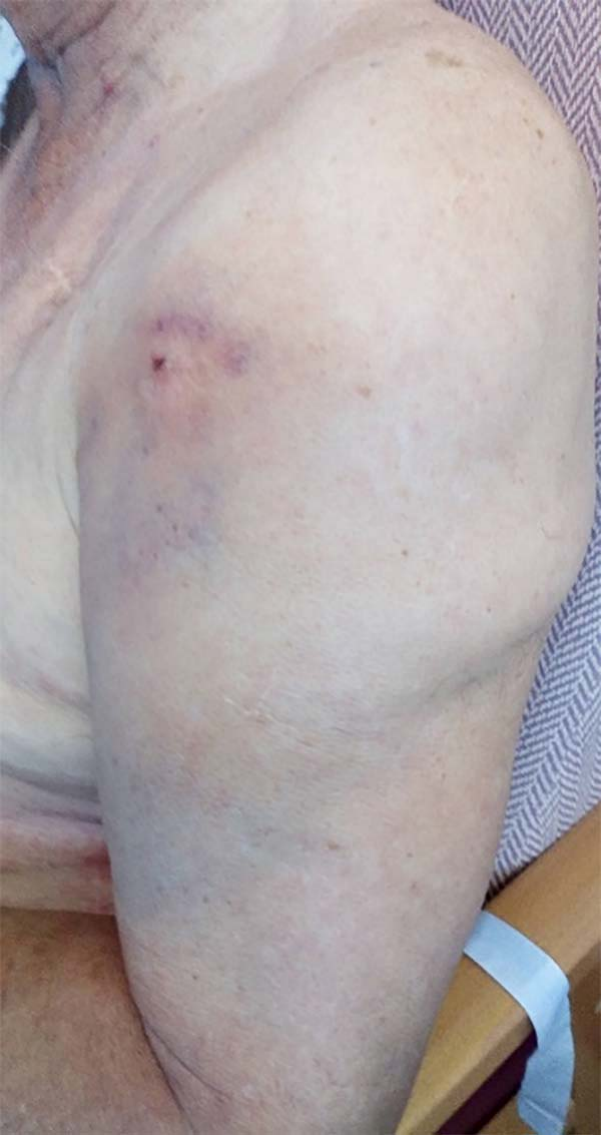
Clinical picture showing un-puckered left shoulder and localized ecchymoses of the anterior arm (lateral view).

However, during pre-operative review on the morning of surgery, the skin was noted to have spontaneously un-puckered (Figs 3 and 4). It appeared viable and there was no break in its integrity.

**Figure 5:**
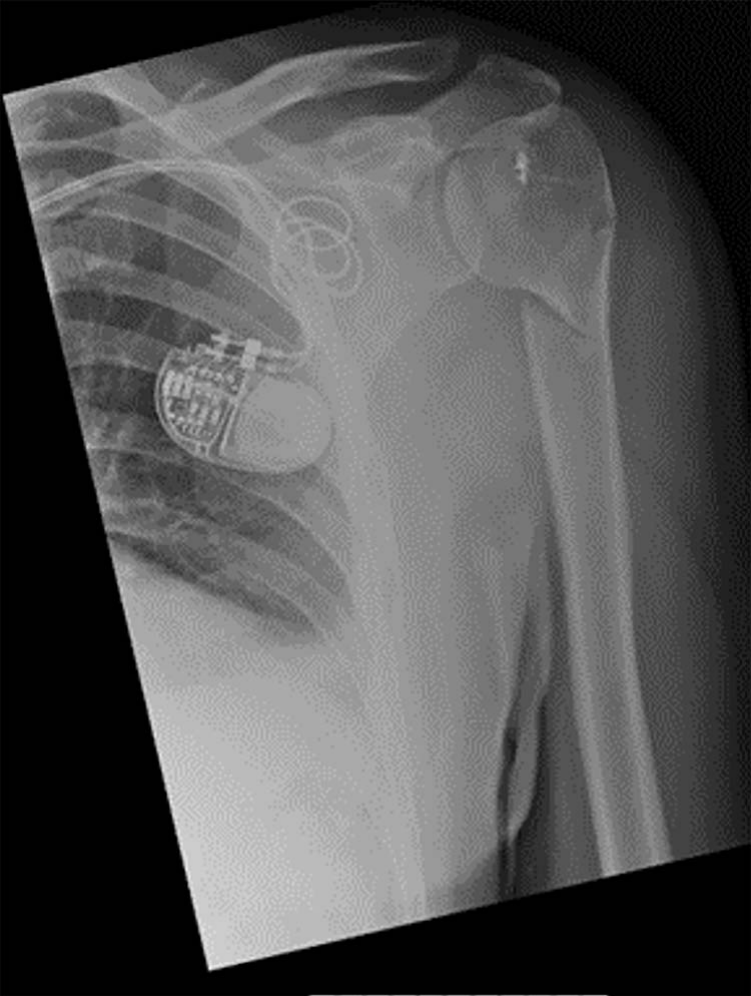
Antero-posterior view X-ray taken 1 week post-injury showing acceptable positioning of the fracture.

**Figure 6:**
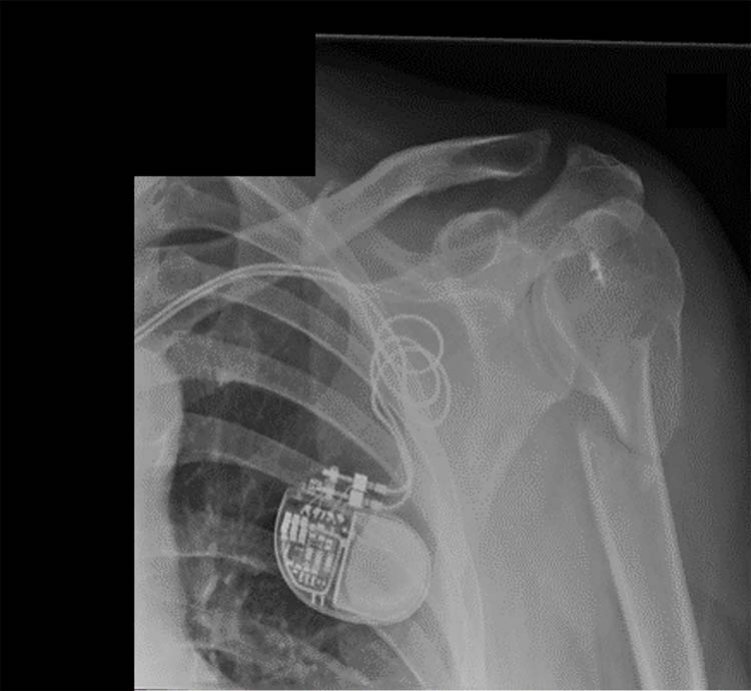
Antero-posterior view X-ray taken 2 weeks post-injury showing acceptable positioning of the fracture.

An X-ray of the left shoulder was performed, which revealed acceptable positioning of the fracture. Following a discussion with the patient, a mutual decision was made to manage the injury non-operatively and he was discharged with a collar and cuff sling. Subsequent clinical and radiological review at 1 week and 2 weeks post-injury showed the soft tissues to be healing well and the fracture to be well-positioned (Figs 5 and 6).

**Figure 7:**
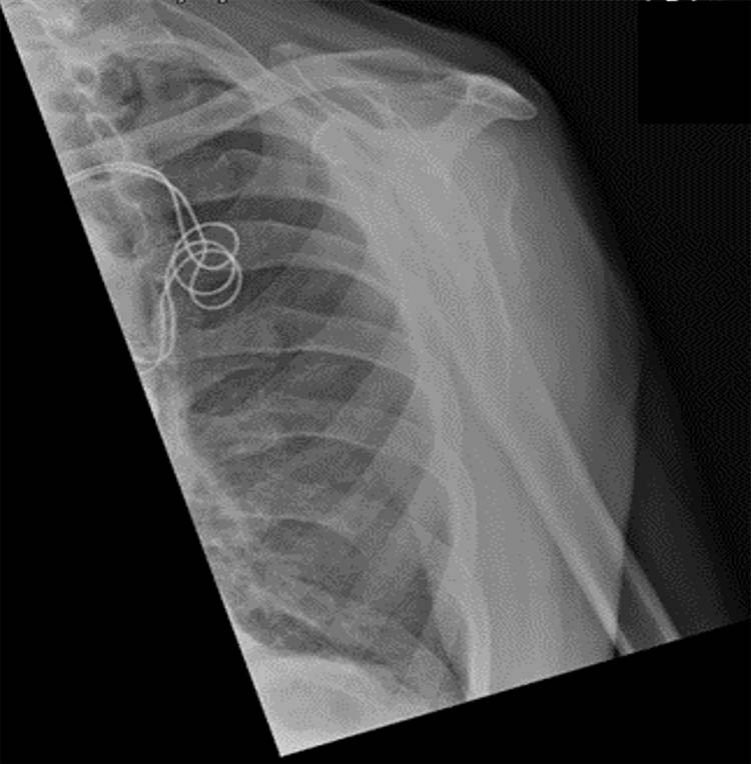
Lateral scapula (Y) view X-ray taken 6 weeks post-injury showing radiological healing of the fracture.

**Figure 8:**
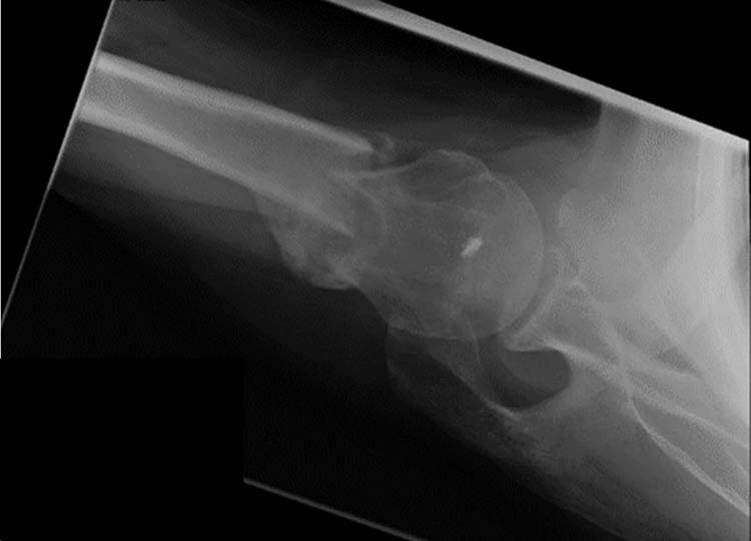
Axial view X-ray taken 14 weeks post-injury showing excellent fracture union and articulation between the humeral head and glenoid.

Review at 6 weeks post-injury demonstrated both clinical and radiological healing (Fig. 7). At a final 14-week review, the patient informed that he had returned to playing golf with no adverse symptoms. Examination revealed complete soft tissue healing over the fracture site and the range of movement was recorded as 120° of both abduction and forward flexion. Plain radiographs confirmed excellent fracture union, and the patient was discharged (Fig. 8).

## DISCUSSION

The ‘Pucker sign’ is a rare and important clinical sign associated with fractures. It often indicates a high risk of soft tissue necrosis and thus necessitates urgent address. Although infrequent, the ‘Pucker sign’ has been reported in fractures of the tibia, clavicle, proximal humerus, supracondylar humeral and distal radius [6]. Alshryda *et al*. were the first to report on skin puckering as a clinical sign in humeral neck fractures. They described their experience with three patients who all required open reduction and internal fixation [1]. The sign has since been reported infrequently.

Fractures of the proximal humerus are the second most common in adults above the age of 65, and orthopaedic trauma services assign a significant part of their workload to the operative management of such injuries [1, 7]. Therefore, understanding the implications of the ‘Pucker sign’ on management and outcomes can be extremely valuable to clinicians.

Patients presenting with a ‘Pucker sign’ are usually offered urgent further intervention. Non-surgical manipulation can be attempted to relieve the soft tissues of tension. However, surgical fixation, which provides fracture stability and soft tissue protection, is often the treatment of choice [8]. Both of these modalities are not without potential harm and must be approached with caution, particularly in more frail and elderly patients. The literature shows that surgical intervention is almost always required in the management of humeral neck fractures with ‘Pucker sign’. However, a 2016 case report provided evidence that gentle axial traction was enough to immediately relieve skin puckering, allowing time to monitor recovery and rearrange surgical repair if required, which in their case, was needed due to ongoing uncontrolled pain [5].

There is a paucity of literature describing self-resolution of skin puckering following fractures of the proximal humerus, which then allowed for successful non-operative treatment. A hypothesis as to why the un-puckering occurs may be as follows: upon diagnosis, immediate management of patients with a surgical neck of humerus fracture includes the application of a collar and cuff sling. This, along with appropriate analgesia, promotes soft tissue relaxation, with subsequent gravitational traction on the distal fracture fragments. Those fragments that had caused skin puckering by piercing the clavipectoral and the deltotrapezial fasciae can then disengage from the skin and subcutaneous tissues, leading to un-puckering and restoration of cutaneous blood supply. If this occurs within 12–24 h of initial injury, the soft tissue envelope will no longer be at risk of being breached secondary to irreversible ischaemia.

To the best of our knowledge, this is the first description of the spontaneous un-puckering of soft tissues overlying a fracture of the proximal humerus. The likelihood of this occurring in patients presenting with a ‘Pucker sign’ is unknown, though rare. However, a safe period of observation of such injuries, where appropriate, is recommended. This may prevent unnecessary patient intervention without causing detriment to the clinical outcome.
